# Exploring Emotional Eating and Emotion Dysregulation in Fibromyalgia Patients: Implications for Disease Management

**DOI:** 10.3390/healthcare14111577

**Published:** 2026-06-04

**Authors:** Mehmet Serhat Topaloğlu, Meltem Puşuroğlu

**Affiliations:** 1 Department of Physical Medicine and Rehabilitation, Faculty of Medicine, Recep Tayyip Erdoğan University, Rize 53100, Türkiye; 2 Department of Psychiatry, Faculty of Medicine, Recep Tayyip Erdoğan University, Rize 53100, Türkiye

**Keywords:** emotional eating, emotion dysregulation, fibromyalgia, psychological symptoms

## Abstract

Background/Objectives: Fibromyalgia (FM) is a complex disease with symptoms such as brain fog, widespread body pain, sleep disturbances, and mood changes, and its etiology is not clearly understood. Our main aim in this study was to evaluate emotional eating and emotional dysregulation in patients with FM and examine the possible effects of these disorders on disease severity. Materials and Methods: This observational study included 94 patients with FM (6 males, 88 females; mean age: 44.64 ± 9.04 years; range, 19–65 years) and 76 controls (7 males, 69 females; mean age: 41.91 ± 10.874 years; range, 18–64 years). The patient and control group participants completed a form including sociodemographic data. Participants also completed the Fibromyalgia Impact Questionnaire (FIQ), Emotional Eating Questionnaire (EEQ), and Difficulties in Emotion Regulation Scale (DERS). Results: In unadjusted comparisons, DERS-goals and DERS-strategies scores were higher in the FM group than in controls; however, these differences did not remain statistically significant after Bonferroni correction. In the linear regression model, it was found that the total score of the Difficulties in Emotion Regulation Scale (DERS-Total) (*p* = 0.010) was the only variable that significantly affected the FIQ value. Even though the patient group had slightly higher EEQ scores, there was no statistically significant difference between the two groups (*p* = 0.721). Conclusions: Emotional regulation difficulties were associated with FM symptom severity, whereas emotional eating did not differ significantly between groups and was not associated with symptom severity. These findings suggest that emotion-related psychological processes should be considered within the biopsychosocial framework of FM.

## 1. Introduction

Fibromyalgia (FM) is a complex disease with symptoms such as brain fog, widespread body pain, sleep disturbances and mood changes, the etiology of which has not been clearly resolved. Many mechanisms are involved in the etiology of the disease. The main causes are central mechanisms, peripheral abnormalities, possible neuroendocrine causes, genetic predisposition, oxidative stress load, and psychosocial causes [1]. When we examine the general population, it is stated that the female population is more affected, but it is seen with a frequency of 2–8% in society [2]. Studies show that FM affects the child population as well as the adult group [3]. FM can be seen in isolation or in association with many psychiatric disorders. The most common of these are depression and anxiety disorders [4]. Recent studies have reported that FM may be accompanied by various psychiatric findings [5]. FM is not merely a pain syndrome affecting the musculoskeletal system. Today, it is defined as a multidimensional condition involving dysfunction in the pain pathways of the central nervous system. The frequent co-occurrence of central pain sensitivity and psychological symptoms underscores the need to address fibromyalgia within a biopsychosocial model. Numerous factors have been implicated in the etiology and pathogenesis of the condition. It has been demonstrated that, in addition to biological processes, psychiatric factors also contribute to the development of the condition [6].

Emotional eating refers to the tendency of individuals to consume food in response to their emotional states. Emotional eating has important physical and psychological effects on health. It is often accompanied by negative emotions, such as anxiety and depression [7]. Stress is an equally important precipitant of eating disorders [8]. In this context, it is thought that negatively perceived conditions such as chronic pain, intense stress, fatigue, and difficulty in emotional regulation observed in FM patients may be related to emotional eating behaviours.

Emotional regulation is the ability of an individual to understand his/her own emotions, set goals and methods to regulate these emotions, and integrate them into his/her life. Emotional regulation problems occur in cases where negative reactions to emotional stimuli are given, emotional awareness is lacking, and the ability to manage emotional expression is limited [9]. One study found that negative emotions increased and positive emotions decreased in FM patients [10]. This may indicate that FM patients have poor emotional regulation skills and that this weakness may be related to pain and other symptoms.

The relationship between FM and mental health conditions has been the subject of increasing research, particularly in recent years. However, a review of the literature shows that FM has been associated primarily with mental health conditions such as depression, fatigue, and insomnia [11]. However, FM is not merely a syndrome that accompanies mental illnesses. The chronic symptoms that often arise can strain patients’ emotional resilience. Individuals who struggle to cope with their emotions using appropriate coping mechanisms may experience the symptoms of the condition more intensely. Individuals whose emotional regulation skills are not fully developed may adopt maladaptive coping strategies. It has been reported that maladaptive coping strategies, such as emotional eating, may be associated with symptom perception and overall health in some individuals [12,13].

Studies have reported that patients with chronic pain exhibit increased eating behaviours for reasons such as distraction, coping with negative emotions, and reducing emotional distress. The underlying cause of these eating behaviours may be coping with negative emotions. In such cases, focusing solely on the pain may prove insufficient for the management and treatment of the condition [14].

FM is a chronic illness involving many complex psychological symptoms. The emotional difficulties experienced by FM patients can affect their coping behaviours. Some individuals may use food consumption as a coping strategy in response to emotional stress. Emotional eating behaviours are reported to be associated with overall health and psychological well-being in various chronic diseases. Our main aim in this study was to evaluate emotional eating and emotional dysregulation in FM patients and to examine the possible effects of these disorders on disease severity. It may also contribute to the development of personalised treatment protocols in FM patients and increase awareness of the management of emotional eating and emotional dysregulation behaviours in patients with FM. In our study, we hypothesised that emotional regulation disorders and emotional eating behaviours may exacerbate FM symptoms. Research on this subject is limited in the literature. Our research results contribute to the literature in this field.

## 2. Materials and Methods

### 2.1. Study Design

This study was conducted between 1 September 2023 and 1 September 2024 at the Physical Medicine and Rehabilitation Outpatient Clinic of Recep Tayyip Erdoğan University Training and Research Hospital. The study was designed as a cross-sectional, observational study.

### 2.2. Participants

It included 94 patients with FM (6 males, 88 females; mean age: 44.64 ± 9.04 years; range, 19–65 years) and 76 controls (7 males, 69 females; mean age: 41.91 ± 10.874 years; range, 18–64 years). The control group consisted of healthy individuals of the same age and sex distribution who had not previously been diagnosed with psychiatric disorders and had no history of neurological disease. The control group was community-based and consisted of relatives of patients who visited the hospital for various reasons, hospital staff members, and individuals undergoing general check-ups; participants were recruited using a sequential sampling method from eligible volunteers who visited the outpatient clinic. The study included patients diagnosed with FM according to the 2016 American College of Rheumatology (ACR) diagnostic criteria. The criteria for participation in the study are defined as being between the ages of 18 and 65, agreeing to participate in the study, having the academic capacity to complete the scale forms, not having a diagnosis of inflammatory rheumatic disease, not having a diagnosis of mental or neurological disease, not having a diagnosis of a chronic pain syndrome other than FM, and not having received pharmacological or non-pharmacological treatment for FM. Individuals in the FM group with a history of major depressive disorder, anxiety disorder, or other psychiatric conditions were excluded from the study. In addition, participants were screened for a history of psychiatric conditions based on their clinical evaluations and self-reports. The assessment of existing psychiatric disorders among participants was conducted through clinical psychiatric interviews, using the Diagnostic and Statistical Manual of Mental Disorders, Fifth Edition (DSM-5) diagnostic criteria and the Structured Clinical Interview for DSM-5 (SCID-5).

### 2.3. Variables and Instruments

Sociodemographic data, body mass index, age, sex, occupation, duration of disease, and marital status were recorded through a form. In addition, the Fibromyalgia Impact Questionnaire (FIQ), Emotional Eating Questionnaire (EEQ) and Difficulties in Emotion Regulation Scale (DERS) were applied to both patient and control groups. The questionnaires were completed by the participants under the supervision of the researchers in an outpatient clinic setting, and the process took approximately 20–25 min. In addition, the participants’ height and weight were measured by the researchers using standard measurement methods during the outpatient clinic evaluation. Body mass index was calculated by dividing body weight in kilograms by the square of height in meters (kg/m^2^). Body mass index (BMI) assessment was conducted according to the World Health Organization classification. Power analysis was performed using the G*Power 3.1 (Heinrich-Heine Universitat Dusseldorf, Dusseldorf, Germany) programme. In the power analysis, the minimum number of people to be included in each group in an analysis with an alpha value of 0.05, an effect size of 0.5, and 80% power was determined to be 64.

#### 2.3.1. Fibromyalgia Impact Questionnaire (FIQ)

The FIQ is a short self-administered assessment tool comprising 10 items, starting with physical functioning in the first section and questioning loss of working day, sleep disturbance, pain, morning stiffness, depression, anxiety, fatigue and general well-being in the other sections. There is a safety and validity study in the Turkish language [15,16]. The Cronbach’s alpha coefficient of the FIQ in the present study was found to be 0.72.

#### 2.3.2. Emotional Eater Questionnaire (EEQ)

The EEQ was developed to assess emotional eating levels. Its Turkish validity and reliability were conducted by Arslantaş et al. Increasing scores are considered as increasing emotional eating levels. It is a self-report scale in which the participant reads and marks the option that they find closest to themselves. It can be applied with internal consistency to the Turkish sample. In our research sample, the Cronbach’s alpha value of the scale was 0.78. In the validity and reliability analysis of the scale, the Cronbach’s alpha value was 0.84 [17,18].

#### 2.3.3. Difficulties in Emotion Regulation Scale (DERS)

This is a Likert-type scale that the participant reads and fills in by himself/herself. It was developed to measure difficulty in emotional regulation. As the scores obtained from the scale increase, it is thought that the difficulty in emotion regulation increases. The Turkish validity and reliability of the scale was conducted by Yiğit et al. The original scale was reorganised as a short form with 16 questions. The DERS consists of the following subscales: clarity, goals, impulse, strategies, and non-acceptance. In our study, all subscales were assessed, and each subscale was analysed and interpreted statistically on its own. In the validity and reliability studies, it was seen that the short form could be used in the Turkish sample. In our research sample, the Cronbach’s alpha value of the scale was 0.82 [19,20].

### 2.4. Ethical Considerations

All participants were adequately informed about the study, and written informed consent was obtained from each individual. Prior to the initiation of the research, ethical approval was obtained from the Non-Interventional Clinical Research Ethics Committee of the Faculty of Medicine at Recep Tayyip Erdogan University (Ethics Approval No: E-71006170-050.01.04-8858; Date: 8 August 2023; Decision No: 2023/175). The study was conducted in accordance with the ethical principles outlined in the 1964 Declaration of Helsinki. Throughout the research process, the ethical standards of the relevant institutional and/or national ethics committees were strictly followed.

### 2.5. Statistical Analysis

Statistical analysis of the research data was performed with the 25th version of the SPSS programme (IBM Corp, Armonk, NY, USA). Descriptive characteristics of the research data were presented as number, minimum, maximum, mean, standard deviation, and percentage values. Chi square and Fisher’s exact test were used to analyse categorical data. The normality of the data was evaluated with the Kolmogorov–Smirnov test, histogram visuals, skewness, and kurtosis values. Independent sample *t*-tests were used to evaluate the mean difference between two groups in the data determined to be compatible with the normal distribution and a one-way analysis of variance (ANOVA) test was used in more than two groups. In case of a significant difference between the groups, the difference between the groups was calculated by post hoc Bonferroni analysis. The correlation of the data was performed with Pearson’s correlation test. Multiple statistical comparisons were made between scales and subscales in the study. In the multiple correlation analyses conducted using age, BMI, and disease duration, as well as in the comparisons of DERS subscales, DERS total, and EEQ scores between the FM and control groups, Bonferroni correction was applied for multiple comparisons, and corrected significance levels of *p* < 0.006 and *p* < 0.007 were accepted, respectively. In accordance with the hypothesis of the study, the FIQ value was taken as the dependent variable, and sex, age, BMI, DERS, and EEQ total score were taken as independent variables, and a linear regression model was established. The established model was statistically significant (F(5, 86) = 2.487; Model *p* = 0.037; Adj. R^2^ = 0.126). Statistical significance was taken as *p* < 0.05.

## 3. Results

A total of 94 patients with FM and 76 healthy controls were included in the study. The youngest patient group was 19 years old and the oldest was 65 years old; the mean age was 44.64 ± 9.04 years. Eighty-eight (93.6%) were female and six (6.4%) were male. The mean body mass index of the patients was 28.05 ± 5.05 kg/m^2^. There was no statistical difference between the sociodemographic data of the patient and control groups including age, sex, body mass index, education, occupation, and marital status (*p* > 0.05) (Table 1). The clinical and sociodemographic data of the patient and control groups are presented in Table 1.

In the patient group, age was negatively correlated with Clarity (r = −0.223, *p* = 0.031); negatively correlated with Goal (*p* < 0.001, r = −0.404); negatively correlated with Impulse (r = −0.210, *p* = 0.043); negatively correlated with Strategies (r = −0. 246, *p* = 0.017); Non-acceptance in a negative direction (r = −0.309, *p* = 0.002); DERS-TOTAL in a negative direction (r = −0.315, *p* = 0.002); and EEQ in a negative direction (r = −0.317, *p* = 0.002). There was a significant negative correlation between body mass index and non-acceptance (r = −0.233, *p* = 0.024). The strategies and non-acceptance scores of university and high school graduates were significantly higher than those of primary school graduates (*p* = 0.013 and *p* = 0.004, respectively). However, after applying the Bonferroni correction, the correlations between age and Goals, Non-acceptance, DERS total score, and EEQ remained significant, while the other significant correlations lost their significance. In addition, the EEQ scores of university graduates were significantly higher than those of primary school graduates (*p* = 0.048), while no difference was found in the EEQ scores of university and high school graduates. In comparison to the patients who were not employed, the EEQ scores of the patients whose occupation was clerk were found to be higher (*p* = 0.010). The relationship between the scale scores of the patients and their sociodemographic and clinical data is presented in detail in Table 2.

When the difference between the scale scores of the patient and control groups was analysed, the Goal and Strategies scores of the patient group were significantly higher than the control group (*p* = 0.048, *p* = 0.029, respectively). However, these differences did not remain statistically significant after Bonferroni correction for multiple comparisons. The difference in the scale scores of the patient and control groups is presented in Table 3.

A linear regression model was established for the evaluation of the factors affecting FM symptom severity. When the model was examined, it was found that the total score of the DERS-TOTAL (*p* = 0.010) was the only variable that significantly affected the FIQ value, and a 1-unit increase in the total score of the DERS led to an increase of 0.289 units in the FIQ value. The other independent variables, age (*p* = 0.903), sex (*p* = 0.054), BMI (*p* = 0.468), and total score of the Emotional Eating Questionnaire (*p* = 0.683), were not found to be effective variables on the FIQ value. Linear regression analysis results for factors associated with FM symptom severity are presented in Table 4.

## 4. Discussion

In our study, the relationship between emotional dysregulation and emotional eating with FM symptom severity in FM patients was investigated. In the unadjusted analyses, the FM group showed higher scores in the Goals and Strategies subscales of the DERS compared with controls; however, these differences did not remain statistically significant after Bonferroni correction. Therefore, these subgroup differences should be interpreted cautiously. In contrast, within the FM group, the DERS-total score was significantly associated with FIQ scores in the linear regression model, suggesting that greater emotional regulation difficulty may be related to higher symptom severity. However, no difference was found between the emotional eating levels of the patient and healthy control groups, and no relationship between emotional eating and symptom severity was found.

FM is characterised by widespread muscle pain, general body tenderness, fatigue, sleep problems, and mental health problems. The predominant symptom in patients is pain. Attempts at controlling this disease involve individually planned treatment programmes including pharmacological and nonpharmacological approaches. The principal aim of treatment is to manage pain, fatigue, sleep problems, and cognitive problems [21]. Pain symptoms are frequently seen in FM patients and depression and anxiety are common in these patients. It is known that alexithymic indicators increase in individuals with chronic pain, such as FM, and these patients have difficulty interpreting their bodily sensations [22]. Although FM has been accepted as a physical disorder, its association with mental disorders has attracted attention, especially recently. Mental symptoms accompanying the current symptoms of patients have revealed the necessity of addressing this patient group from a psychiatric perspective. Symptoms such as depressive disorder and anxiety disorder may frequently accompany the current symptoms of the patients. However, other mental symptoms other than these diagnoses have an important place in the disease’s prognosis. Patients’ emotional management, emotional expression, and acceptance of their emotions can be effective for symptoms. Given the biopsychosocial nature of FM, it is believed that emotional regulation processes may be associated with the perception of chronic pain, the stress response, and symptom severity. In particular, it has been reported that the emotional burden accompanying chronic pain may interact with central sensitisation processes and psychological stress mechanisms. The difficulties experienced by patients in managing their emotions also negatively affect the course of the disease. It is observed that FM patients have difficulties in emotional management, cannot control their emotions, and have more difficulty managing emotions such as anger [23]. Although the FM group tended to show higher scores in some DERS subdomains in unadjusted analyses, these differences did not survive correction for multiple comparisons. Nevertheless, the significant association between DERS-total and FIQ scores suggests that emotional regulation difficulties may be clinically relevant to symptom severity among patients with FM. Consistent with our findings, a study by Trucharte et al. also reported that difficulties with emotion regulation are more pronounced in patients with FM. Furthermore, an association between difficulties with emotional regulation and pain intensity has been demonstrated [24]. Similarly, in a study conducted with a large sample, the relationship between the emotional regulation difficulties of FM patients and pain symptoms was examined. In this study, a relationship was found between emotional regulation difficulties and symptom severity. In addition, it was stated that patients with higher pain severity had higher levels of depression and their quality of life decreased [12]. In a study conducted by Geenen et al. it was shown that emotional regulation difficulties were associated with FM severity, in line with our findings. It was stated that an intense emotional experience and inability to manage emotions may be associated with increased symptom severity and FM may have a negative effect on emotion management [25]. Difficulties with emotional regulation may be related to individuals’ coping strategies. It has been reported that FM patients who use negative problem-solving strategies may experience greater symptom severity and a higher prevalence of psychiatric symptoms such as depression and anxiety [26].

Emotional avoidance behaviour and negative coping strategies towards emotions are associated with more symptoms such as pain and fatigue in patients [27,28]. Reviewing emotional management and coping strategies during the treatment process may contribute to symptom management. Supportive approaches aimed at helping patients recognise and express their emotions may be beneficial for some patients. There is a need for studies with larger sample sizes to support the potential effects of emotion-focused approaches in the treatment of FM [29,30]. Cognitive-behavioural therapy, acceptance and commitment therapy, and mindfulness-based approaches may be used more frequently in the treatment of pain in patients with FM. Adding emotion-focused psychotherapy to physical-focused treatments may have a positive impact on patients’ prognosis. Most of these approaches focus on emotional regulation, emotion recognition, and the development of coping strategies. Research has shown that these approaches are effective for patients with FM [31,32]. In particular, it is thought to influence the interaction between chronic pain, the stress response, and emotional regulation processes. Within the biopsychosocial framework of FM, it has been reported that psychological processes may play a role in symptom perception and the experience of the illness. In our study, in addition to emotional regulation difficulties, body mass index, the emotional eating level of the patients, and the relationship between emotional eating and symptom severity were also examined. No difference was found between the emotional eating levels and body mass index values of the patient group and the healthy control group. However, when we look at the literature, there are studies reporting that FM patients have an increased frequency of obesity and eating disorders compared to the healthy population. In a study the body mass index of FM patients was found to be higher than the healthy control group. In addition, eating attitudes of FM patients were found to be more impaired than healthy controls. It was also found that the impaired eating attitudes of patients were associated with symptoms such as depression and anxiety [33]. The literature reports varying findings regarding eating behaviours in patients with FM; however, the results of our study do not support these findings. It is believed that this discrepancy may be influenced by sample characteristics, psychiatric comorbidities, and sociocultural differences. In FM patients, excess weight may adversely affect the symptoms of the patients. In particular, the restriction of movement caused by weight may decrease the quality of life of the patients. Mental symptoms may be observed due to the negative impact on daily life [34]. When the relationship between FM and eating disorders is examined, it has been found that nutrition in particular has a positive effect on mental disorders in FM patients. It is thought that a quality and healthy diet can be both curative in terms of FM symptoms and protective in terms of mental health [35]. In a study conducted with FM patients, eating disorders and obesity were found to increase depressive symptoms. Although no direct relationship between eating disorders and symptom severity was found in patients, it may lead to an indirect negative effect by increasing the frequency and severity of other mental illnesses. However, our study did not find a significant association between emotional eating behaviours and disease severity; therefore, the relationship between nutrition and mental health needs to be evaluated through prospective studies with larger sample sizes [36]. Although emotional eating levels were not found to be different from the healthy control group in our study, emotional eating was found to be higher than that of the healthy control group in a study conducted with FM patients [13]. Similarly, a study conducted in FM patients found that impaired eating habits and inflammatory diet increased FM symptom severity [37]. It is believed that these differences between studies may stem from variations in sample characteristics, psychiatric comorbidities, lifestyle factors, and dietary habits. Furthermore, due to the heterogeneous clinical nature of FM, it has been reported that psychological and behavioural processes may manifest differently among patients. In FM patients, a multidimensional approach to the patient is determined rather than a specific treatment. At this point, a multidisciplinary approach has an important place in the treatment of patients. Treatment strategies, such as nutritional diet programmes and exercise, are important treatment methods. Focusing only on the patient’s physical complaints and ignoring mental symptoms may negatively affect the treatment. In addition, healthy eating habits, regular exercise, and increased stress coping methods have a positive effect on the treatment process [38]. Our research findings suggest that emotion-focused approaches may be effective in the treatment algorithm for FM. Our findings support a possible relationship between emotional regulation difficulties and FM symptom severity, but this association should be interpreted within the limitations of the cross-sectional design and the modest explanatory power of the regression model. In this regard, incorporating psychotherapeutic approaches such as emotional awareness and cognitive reappraisal into cognitive-behavioural and mindfulness-based approaches may enhance the effectiveness of treatment.

This study had certain limitations. Firstly, the research was conducted at a single centre and included participants from a single geographic region. This may have resulted in a homogeneous sample in terms of demographic, cultural, and socioeconomic characteristics, limiting the generalisability of the findings to broader population. Secondly, the cross-sectional design of the study does not reveal the temporal dimension of the relationships between variables or make causal inferences. Therefore, it is not possible to interpret the identified relationships as cause-and-effect relationships. Additionally, potential confounding variables, such as physical activity level, exercise habits, socioeconomic status, level of social support, lifestyle characteristics, and psychosocial stressors, which are known to affect FM symptom severity, were not evaluated in the study. A significant limitation of our study is that dietary habits associated with emotional eating were not examined separately and systematically in relation to physical activity. Future studies could be designed to be more comprehensive by focusing on these factors. Consequently, the fact that these factors were not measured may have created uncontrolled effects on the observed findings. In addition, the fact that the data were largely collected through self-report scales means they may be subject to measurement biases, such as recall bias and response tendencies. The lack of support from clinical assessments or objective measurements is another limitation that should be considered when interpreting these findings.

Finally, the relatively limited sample size may have reduced the study’s statistical power, particularly in the subgroup analyses, and may have prevented the detection of some potential associations. In light of these limitations, future studies should be conducted using larger samples and multi-centre and longitudinal designs. They should also be planned to include biological, psychosocial, and lifestyle variables that may affect FM symptoms, thereby increasing the reliability and generalisability of the findings.

## 5. Conclusions

Although between-group differences in DERS subscales did not reach statistical significance after correction for multiple comparisons, within the FM group, greater difficulties in emotional regulation were significantly associated with higher disease severity, as evidenced by the regression analysis. However, no significant difference was found between the patient and control groups in terms of emotional eating behaviours. Given the biopsychosocial nature of FM, it is believed that psychological processes should also be taken into account when evaluating patients. Many factors such as genetics, early period schemas, and traumatic life events play a role as factors triggering the development of FM. Therefore, a multidimensional evaluation of patients and a multidisciplinary approach will have a positive effect on the disease process. Our study draws attention to the importance of mental symptoms, especially emotion-focused symptoms, when assessing the severity of illness in FM patients. It is important that emotion-orientated symptoms of patients should not be ignored in the follow-up process.

## Figures and Tables

**Table 1:** Comparison of sociodemographic data of patients and control group.

Variables		FM (*n* = 94)		Control (*n* = 76)		Test st.	*p*
		Min–Max	Mean ± SD	Min–Max	Mean ± SD		
Age (years)		19–65	44.64 ± 9.04	18–64	41.91 ± 10.874	1.787	0.076
BMI (kg/m^2^)		19.47–40.26	28.05 ± 5.05	21.19–36.98	26.75 ± 3.35	1.918	0.057
Disease duration (month)		6–180	67.23 ± 49.08				
FIQ		31.44–90.26	62.12 ± 16.21				
		*n*	%	*n*	%	Test st.	*p*
Sex	Female	88	93.6	69	90.8	0.476	0.490
	Male	6	6.4	7	9.2		
Education Level	Primary School	24	25.5	10	13.2	4.033	0.133
	High School	42	44.7	39	51.3		
	University	28	29.8	27	35.5		
Occupation	Not working	67	71.3	49	64.5	3.628	0.163
	Clerk	12	12.8	18	23.7		
	Worker	15	16.0	9	11.8		
Marital Status	Married	75	79.8	58	76.3	3.423	0.181
	Single	8	8.5	15	19.7		
	Widowed	6	6.4	2	2.6		
	Divorced	5	5.3	1	1.3		

Independent sample *t*-test, chi square test, Fisher’s exact test *p* < 0.05. BMI: Body mass index, SD: Standard deviation, FM: Fibromyalgia, FIQ: Fibromyalgia Impact Questionnaire.

**Table 2:** The relationship between patients’ sociodemographic and clinical data and scale scores.

Variables		D1		D2		D3		D4		D5		DERS TOTAL		EEQ		FIQ	
		r	*p*	r	*p*	r	*p*	r	*p*	r	*p*	r	*p*	r	*p*	r	*p*
Age		−0.223	0.031	−0.404	<0.001	−0.210	0.043	−0.246	0.017	−0.309	0.002	−0.315	0.002	−0.317	0.002	−0.066	0.528
BMI		−0.114	0.274	−0.075	0.471	−0.073	0.485	−0.087	0.407	−0.233	0.024	−0.130	0.211	0.106	0.314	0.053	0.612
Disease Duration		−0.003	0.979	−0.010	0.920	0.026	0.806	−0.069	0.510	−0.071	0.497	−0.037	0.723	−0.154	0.142	0.113	0.278
		mean ± SD	*p*	mean ± SD	*p*	mean ± SD	*p*	mean ± SD	*p*	mean ± SD	*p*	mean ± SD	*p*	mean ± SD	*p*	mean ± SD	*p*
Sex	Female (*n* = 88)	4.1 ± 2.1	0.748	8.7 ± 3.2	0.886	6.1 ± 3.5	0.507	10.7 ± 5.5	0.701	6.1 ± 3.6	0.508	35.9 ± 15.9	0.657	8.5 ± 6.4	0.237	65.3 ± 16.0	0.089
	Male (*n* = 6)	4.5 ± 3.5		8.5 ± 4.5		7.1 ± 3.6		11.6 ± 7.3		7.1 ± 5		39 ± 23.2		11.8 ± 5.6		53.6 ± 18.4	
Education Level	Primary School (*n* = 24)	3.9 ± 2.2	0.532	7.7 ± 2.9	0.247	5 ± 3	0.149	7.9 ± 3.6 ^a^	0.013	4 ± 1.7 ^c^	0.004	28.6 ± 10.7	0.052	6.2 ± 4.3 ^e^	0.048	63.5 ± 19.0	0.528
	High School (*n* = 42)	4.5 ± 2.2		9.1 ± 3.3		6.5 ± 3.3		11.6 ± 5.7 ^b^		6.6 ± 3.7 ^d^		38.5 ± 16		9 ± 7 ^e,f^		66.7 ± 14.2	
	University (*n* = 28)	4 ± 2.3		8.7 ± 3.6		6.7 ± 4.2		12 ± 6.3 ^b^		7.2 ± 4.3 ^d^		38.8 ± 19.1		10.6 ± 6.5 ^f^		62.4 ± 17.0	
Marital Status	Married (*n* = 75)	4 ± 2.1	0.220	8.7 ± 3.4	0.884	6.1 ± 3.5	0.840	10.6 ± 5.5	0.436	6 ± 3.7	0.573	35.5 ± 16.3	0.771	8.3 ± 6	0.277	63.0 ± 16.6	0.228
	Single (*n* = 8)	4.5 ± 2.2		8.8 ± 3.6		6.2 ± 4.3		8.8 ± 4.7		6 ± 2.7		34.5 ± 15		8.7 ± 7.2		68.1 ± 15.3	
	Widowed (*n* = 6)	6 ± 1.8		9.1 ± 2.7		6.5 ± 3.2		13 ± 6.1		6.6 ± 3.5		41.3 ± 14.2		13.6 ± 9		75.7 ± 9.2	
	Divorced (*n* = 5)	3.8 ± 3		7.6 ± 3.7		7.6 ± 4		13.2 ± 8.2		8.4 ± 5.5		40.6 ± 23		9.8 ± 7.8		69.6 ± 16.3	
Occupation	Not working (*n* = 67)	4.2 ± 2.2	0.705	8.7 ± 3.1	0.766	6 ± 3.1	0.458	10.4 ± 5.2	0.192	5.8 ± 3.2	0.150	35.3 ± 14.8	0.435	7.5 ± 5.8 ^g^	0.010	65.7 ± 15.8	0.429
	Clerk (*n* = 12)	3.7 ± 2.3		9 ± 4		7.4 ± 5.3		13.5 ± 7.7		8 ± 5.4		41.8 ± 22.9		12.7 ± 6.5 ^h^		59.1 ± 14.1	
	Worker (*n* = 15)	4.4 ± 2.4		8.1 ± 3.6		5.8 ± 3.5		10.3 ± 5.6		6.3 ± 3.9		35.1 ± 17.1		11.2 ± 7.3 ^g,h^		63.9 ± 20.0	

Pearson’s correlation, independent sample *t*-test, one-way ANOVA, *p* < 0.05, Bonferroni correction were applied, Adj. *p* < 0.006. a, b, c, d, e, f, g, h: difference in grouping. DERS: Difficulties in Emotion Regulation Scale; FIQ: Fibromyalgia Impact Questionnaire; EEQ: Emotional Eater Questionnaire. Subscales of the DERS: D1: Clarity; D2: Goals; D3: Impulse; D4: Strategies; D5: Non-acceptance.

**Table 3:** Comparison of scale scores of the patient and control groups.

Scales	FM (*n* = 94)	Control (*n* = 76)	Test st.	*p*
	Mean ± SD	Mean ± SD		
D1	4.2 ± 2.2	3.9 ± 1.6	0.892	0.374
D2	8.6 ± 3.3	7.7 ± 2.6	1.990	0.048
D3	6.2 ± 3.5	5.4 ± 2.1	1.771	0.078
D4	10.8 ± 5.6	9.2 ± 3.7	2.201	0.029
D5	6.1 ± 3.7	5.8 ± 2.7	0.764	0.446
DERS-TOTAL	36.1 ± 16.3	32.1 ± 9.8	1.947	0.053
EEQ	8.8 ± 6.4	8.4 ± 5.5	0.357	0.721

Independent sample *t*-test, *p* < 0.05, Bonferroni correction was applied Adj. *p* < 0.007. DERS: Difficulties in Emotion Regulation Scale; EEQ: Emotional Eater Questionnaire; FM: Fibromyalgia. Subscales of the DERS; D1: Clarity; D2: Goals; D3: Impulse; D4: Strategies; D5: Non-acceptance.

**Table 4:** Linear regression model of factors affecting fibromyalgia symptom severity.

Variables	Unstandardised Coefficient (95% CI)	SE	Standardised Coefficient	t	*p*	Zero	Partial	Part	VIF
(Constant)	32.259 (3.565–60.953)	14.434		2.235	0.028				
Age	0.026 (−0.402–0.455)	0.216	0.015	0.122	0.903	−0.064	0.013	0.012	1.411
Sex (Female)	13.165 (−0.241–26.572)	6.744	0.199	1.952	0.054	0.179	0.206	0.197	1.020
BMI	0.264 (−0.456–0.984)	0.362	0.082	0.729	0.468	0.052	0.078	0.073	1.247
DERS-TOTAL	0.289 (0.071–0.507)	0.110	0.290	2.638	0.010	0.282	0.274	0.266	1.193
EEQ	0.120 (−0.461–0.701)	0.292	0.047	0.410	0.683	0.121	0.044	0.041	1.299

F(5, 86) = 2.487; Model *p* = 0.037; Adj. R^2^ = 0.126; SE of Estimate = 15.817; Durbin-Watson = 1.966. DERS: Difficulties in Emotion Regulation Scale, EEQ: Emotional Eater Questionnaire, BMI: Body mass index.

## Data Availability

The datasets used and/or analysed in the present study are available from the corresponding author upon reasonable request.
